# Impact of a Multistrain Probiotic Formulation with High Bifidobacterial Content on the Fecal Bacterial Community and Short-Chain Fatty Acid Levels of Healthy Adults

**DOI:** 10.3390/microorganisms8040492

**Published:** 2020-03-30

**Authors:** Giorgio Gargari, Valentina Taverniti, Ranjan Koirala, Claudio Gardana, Simone Guglielmetti

**Affiliations:** 1 Division of Food Microbiology and Bioprocesses, Department of Food, Environmental and Nutritional Sciences, University of Milan, 20133 Milan, Italy; gargari.g@gmail.com (G.G.); valentina.taverniti@unimi.it (V.T.); ranjan.koirala@unimi.it (R.K.); 2 Division of Human Nutrition, Department of Food, Environmental and Nutritional Sciences, University of Milan, 20133 Milan, Italy; claudio.gardana@unimi.it

**Keywords:** DESeq2, ALDEx2, short-chain fatty acids, succinate, acetate, butyrate, lactate, fecal microbiota, intervention trial

## Abstract

The consumption of probiotic products is continually increasing, supported by growing scientific evidence of their efficacy. Considering that probiotics may primarily affect health (either positively or negatively) through gut microbiota modulation, the first aspect that should be evaluated is their impact on the intestinal microbial ecosystem. In this study, we longitudinally analyzed the bacterial taxonomic composition and organic acid levels in four fecal samples collected over the course of four weeks from 19 healthy adults who ingested one capsule a day for two weeks of a formulation containing at least 70 billion colony-forming units, consisting of 25% lactobacilli and 75% *Bifidobacterium animalis* subsp. *lactis*. We found that 16S rRNA gene profiling showed that probiotic intake only induced an increase in a single operational taxonomic unit ascribed to *B. animalis*, plausibly corresponding to the ingested bifidobacterial strain. Furthermore, liquid chromatography/mass spectrometry revealed a significant increase in the lactate and acetate/butyrate ratio and a trend toward a decrease in succinate following probiotic administration. The presented results indicate that the investigated probiotic formulation did not alter the intestinal bacterial ecosystem of healthy adults and suggest its potential ability to promote colonization resistance in the gut through a transient increase in fecal bifidobacteria, lactic acid, and the acetate/butyrate ratio.

## 1. Introduction

The consumption of products containing probiotics, i.e., “live microorganisms that, when administered in adequate amounts, confer a health benefit on the host” [1], is constantly increasing worldwide, supported by the growing scientific evidence of their efficacy [2]. According to the scientific literature and existing regulatory approaches, a positive effect can be obtained when probiotics are ingested in a quantity of at least 1 billion microorganisms per day (Italian Ministry of Health) or per serving (Health Canada) [1]. Nonetheless, several studies have suggested that some beneficial effects can only be obtained by consuming much higher doses of probiotic cells [3,4,5]. In this context, a human intervention trial with two doses (7 and 70 billion) of a multispecies probiotic formulation revealed that the higher dose allowed an early and prolonged recovery, thus indicating a potential greater permanence of probiotic cells in the gastrointestinal tract [6].

Consistent with the observation that higher doses can be more effective than lower doses and due to improved industrial technologies for microbial biomass production and preservation, in recent years, the market has seen the steady growth of probiotic formulations with an increased number of viable microbial cells, reaching up to hundreds of billions of colony-forming units (CFUs) per single dose. However, the ingestion of such amounts of microbial cells is not unprecedented. For example, approximately one hundred billion live bacteria can be ingested by consuming 100 g of yogurt or other traditional fermented products (kimchi, sauerkraut, etc.). Nonetheless, the abovementioned examples refer exclusively to the ingestion with food of lactic acid bacteria, whereas probiotic formulations often contain bifidobacteria, common inhabitants of the mammalian gut, which have been extensively documented to provide numerous different health benefits to the human host [7]. In particular, the bifidobacteria most commonly included in commercial probiotic formulations worldwide belong to *Bifidobacterium animalis* subsp. *lactis*, a taxon constituted by closely related and isogenic strains [8], whose probiotic properties have been investigated in several hundred scientific studies; these studies have shown that these bacteria possess the ability to decrease intestinal transit time [9] and improve constipation [10,11,12], prevent antibiotic-associated diarrhea [13,14], enhance innate immunity markers in older people [15], improve outcomes related to gastrointestinal discomfort in healthy adults [16], and alleviate atopic eczema symptoms in infants [17].

Bifidobacteria are not naturally present in food, except in small numbers and rare cases of exceptions. One example of a food product containing bifidobacteria in its natural microbiota is kumis (or airag), a Central Asian product made by fermenting raw unpasteurized mare’s milk that contains approximately 6 million CFUs of bifidobacterial cells per ml [18]. Therefore, unlike several types of lactic acid bacteria, viable bifidobacteria are not traditionally consumed in food, especially not in the amounts consumed through the ingestion of modern probiotic formulations. It therefore appears necessary to assess whether the intake of bifidobacteria can be considered safe even in higher quantities. Particularly, considering that probiotics may perform part of their activity on the host by modulating the gut microbiota, a first aspect that should be evaluated is the possible impact on the intestinal microbial ecosystem exerted by probiotics containing a high number of viable bifidobacterial cells (here, arbitrarily considered to be higher than 5 × 10^10^). To this aim, in this study, we longitudinally analyzed the taxonomic composition of the fecal microbiota and short-chain fatty acid levels in 19 healthy adults who ingested one capsule a day for two weeks of a probiotic formulation containing 7 × 10^10^ CFUs, 25% of which were lactobacilli, and 75% of which were *Bifidobacterium animalis* subsp. *lactis*.

## 2. Materials and Methods

### 2.1. Microbiological Composition of the Probiotic Formulation

The probiotic product studied here consisted of methylcellulose capsules with a declared microbial concentration of 7 × 10^10^ CFUs, available on market in Italy with the commercial name “FlorMidabìl™”. The formulation was made of a blend of four bacterial strains: *B. animalis* subsp. *lactis* Bl-04 (nominal concentration of 5.25 × 10^10^ CFU per capsule), *L. acidophilus* La-14 (1.4 × 10^10^ CFU per capsule), *L. plantarum* SDZ-11 (2.8 × 10^9^ CFU per capsule) and *L. paracasei* SDZ-22 (7 × 10^8^ CFU per capsule). In addition, the capsules contained the following excipients: hydroxypropyl methylcellulose (coating agent), microcrystalline cellulose (bulking agent), magnesium salts of fatty acids and silicon dioxide (anti-caking agents), and titanium dioxide (coloring agent).

### 2.2. Intervention and Study Scheme

**Figure 1:**
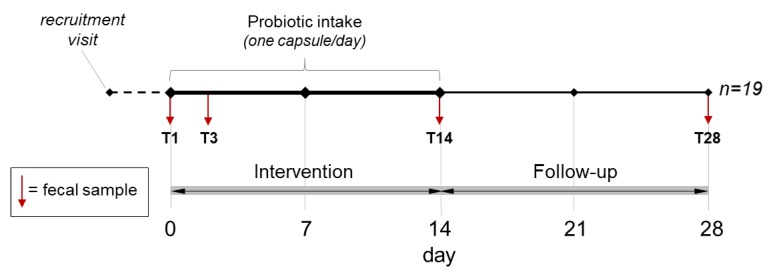
Design of the trial. Vertical arrows indicate the collection and analysis of fecal samples in this study. T3 corresponds to the fecal sample collected between days 3 and 5, depending on the subject (day 3 for most of the volunteers).

The trial consisted of a single-blind microbiological pilot study. The inclusion and exclusion criteria were described in a previous publication [6]. The study protocol was approved by the Research Ethics Committee of the University of Milan (opinion no. 50/17, 18 December 2017). The trial consisted of a two-week intervention (one probiotic capsule per day) followed by two weeks of follow-up. Participants consumed the capsule with natural water in the morning, at least 30 min before breakfast, or in the evening, at least 2 h after the last meal. Four specimens were collected from each of the 19 healthy adults (mean age 29.1 ± 8.8 years) of both sexes (12 females) according to the protocol shown in Figure 1.

### 2.3. Assessment of Bacterial Cell Viability in Probiotic Capsules

Total and viable cells in probiotic capsules were quantified by flow cytometry, as previously described [6]. In brief, the powder in the probiotic capsule was dissolved in Mitsuoka buffer (pH 6.5), homogenized in a sterile bag by using a stomacher (Colworth Stomacher 400, Seward, West Sussex, UK), serially diluted in the same buffer, and stained with the non-permeant red-fluorescent dye propidium iodide and the cell-permeant green fluorescent dye, SYTO™ 24 (15 min incubation at 37 °C in the dark). After staining, samples were immediately analyzed with a C6 BD Accuri™ flow cytometer (BD Biosciences, San Jose, CA, USA; threshold settings: FSC-H: 5000 and SSC-H 2000; total volume collected, 50 µL). The absolute flow cytometry count was carried out using Fluoresbrite polychromatic red 2.0 mm microspheres as a reference.

### 2.4. Fecal Microbiota Taxonomic Profiling

Total DNA was extracted from fecal samples as previously described [6,19]. In brief, we used the DNeasy PowerLyzer PowerSoil DNA extraction kit (Qiagen, Hilden, Germany) following the manufacturer’s instructions with the only difference of incubating samples at 65 °C for 10 min after the addition of C1 solution. The cell disruption step was performed with a Precellys bead beater (3 cycles of 6800 rpm × 30 s; Advanced Biotech Italia s.r.l., Seveso, Italy). Finally, DNA was analyzed at the Institute for Genome Sciences (University of Maryland, School of Medicine, Baltimore, MD, USA) through 16S rRNA gene profiling with Illumina HiSeq 2500 rapid run sequencing of the V3-V4 variable region. Pairing, filtering, taxonomic assignment, and taxonomic diversity analyses of raw amplicon sequencing data were carried out by means of the bioinformatic pipeline Quantitative Insights Into Microbial Ecology (QIIME) version 1.9.0 [20] with the GreenGenes database (version 13_5). FASTQ data have been deposited in the European Nucleotide Archive (ENA) of the European Bioinformatics Institute under accession code PRJEB36929.

### 2.5. Quantification of Organic Acids in Fecal Samples

Organic acids were quantified in feces by ultra-high-pressure liquid chromatography coupled with high-resolution/high-accuracy mass spectrometry (UPLC-HR-MS) as previously described [21,22]. In brief, 100 mg of feces was extracted with 2 mL of 0.001% formic acid. Then, UPLC-HR-MS analysis was performed on an Acquity UPLC separation module (Waters, Milford, MA, USA) coupled with an Exactive Orbitrap MS through a HESI-II probe for electrospray ionization (Thermo Scientific, San Jose, CA, USA). Finally, the UPLC eluate was analyzed by full scan MS in the 50–130 m/z range. Five-point external calibration curves were prepared for the quantification of acetic, butyric, lactic, propionic, succinic, and valeric acids.

### 2.6. Statistical Analysis

Statistical analysis of data was carried out using R statistic software (version 3.4.2). Data from taxonomic diversity analysis were analyzed by the Wilcoxon-Mann-Whitney test. Significantly different bacterial taxonomic units between time points were determined using differential gene expression analysis based on the negative binomial distribution method (R/Bioconductor DESeq2 package; [23]) and the ANOVA-Like Differential Expression tool (R/Bioconductor ALDEx2 package; [24]) followed by Welch’s t-test. In both analyses, *p* values were FDR-adjusted [23] (0.05 was used as the threshold for significance). SCFA quantification data were analyzed by paired Student’s t-test with a *p* < 0.05 significance level, whereas 0.05 < *p* < 0.10 was considered to indicate a trend. Correlation analysis between bacterial relative abundance and SCFA concentration was performed using Spearman’s ρ test.

## 3. Results

### 3.1. Content of Viable Microbial Cells in the Probiotic Formulation

Nineteen capsules of the probiotic product under study (one per volunteer) were randomly selected and analyzed by flow cytometry to quantify the total and viable numbers of cells. Staining of bacterial cells with SYTO™ 24 and propidium iodide revealed a total concentration of 5.08 (± 0.34) × 10^11^ cells per capsule, with a viable count of 2.03 (±0.13) × 10^11^ cells per capsule, which corresponds to a viable count 2.9-times greater than that indicated on the label (70 billion = 7 × 10^10^ CFUs per capsule). This discrepancy between label specifications and the actual concentration of viable cells in the product is expected, given that manufacturers of probiotic products apply an overdosage of live microorganisms in the formulations to ensure that the number of surviving microbial cells is not lower than that specified on the label until the end of the product’s shelf life.

### 3.2. Biodiversity of the Fecal Bacterial Community During the Probiotic Intervention

We used 16S rRNA gene profiling to characterize the bacterial taxonomic composition of 76 fecal samples collected from 19 participants in the study. MiSeq sequencing generated 3,693,607 filtered paired-end reads (mean of 62,603 reads per sample). The average quality per read (phred score) was 37. The sequence length was between 301 and 596 bp, with an average of 460 bp. Overall, we identified 2525 unique operational taxonomic units (OTUs), with a mean and median number of 1019 and 967 per subject, respectively.

**Figure 2:**
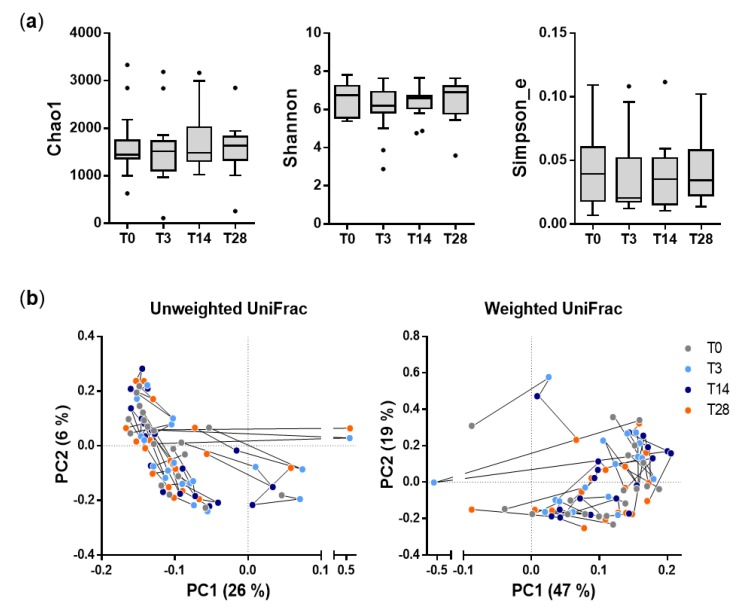
Diversity analysis of fecal bacterial communities during the probiotic intervention trial. (**a**) α-diversity analysis; the panel shows three indexes that differently describe the richness and evenness of bacterial communities in fecal samples. (**b**) β-diversity analysis based on principal coordinate analysis of unweighted (left) and weighted (right) UniFrac distances. Each point in the graph represents a different fecal sample. Colors indicate the different phases of the study, according to the legend on the right. Black lines connect samples from the same subject. The percentages indicate the proportions of variance explained by the first and second ordination axes.

The analysis of α-diversity revealed that the probiotic intervention did not significantly affect the taxonomic richness or evenness of the fecal microbiota structure (Figure 2A). To measure intersample (β-)diversity, we examined the sequence reads with principal coordinate analysis based on weighted and unweighted UniFrac distances. This analysis showed that the overall fecal bacterial community structure was not significantly different among the four phases of the study (Figure 2B).

Overall, these results indicate that the intake of the probiotic formulation did not significantly alter the fecal bacterial community structure of the healthy adults participating in the trial.

### 3.3. Effect of the Probiotic Intervention on the Abundance of Fecal Bacterial Taxa

**Figure 3:**
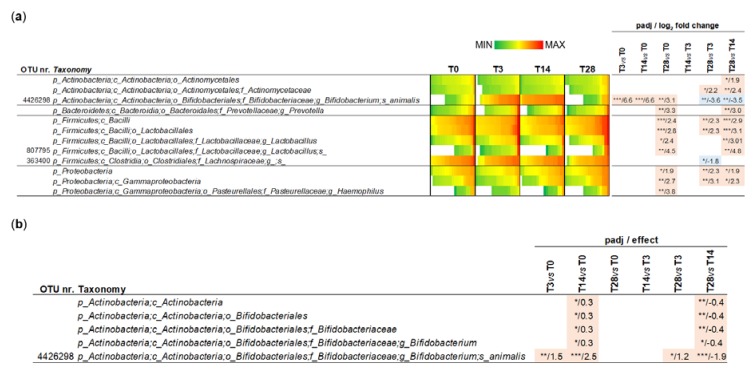
Significantly different operational taxonomic units (OTUs) determined with DESeq2 (**a**) and ALDEx2 (**b**) normalization. Asterisks refer to FDR-adjusted *p* values (padj) from Welch’s Test (*** *p* < 0.001; ** *p* < 0.01; * *p* < 0.05).

**Figure 4:**
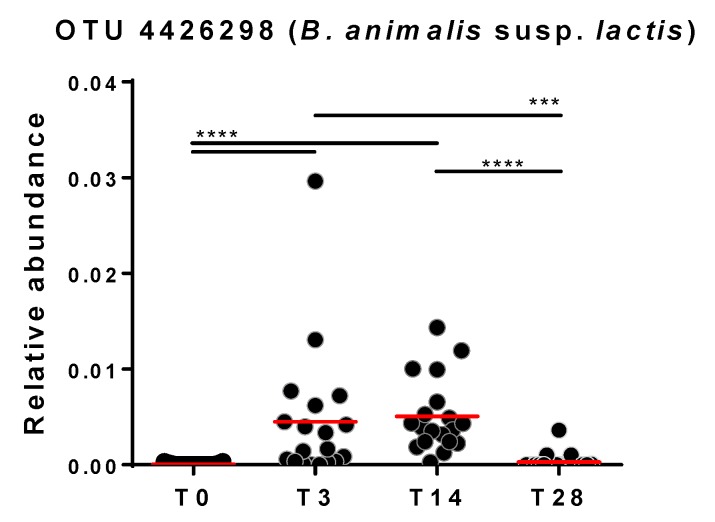
Relative abundance of OTU X4426298 over the course of the probiotic intervention trial (OTU taxonomy: *p_Actinobacteria; c_Actinobacteria; o_Bifidobacteriales; f_Bifidobacteriaceae; g_Bifidobacterium; s_animalis*). Statistics according to Wilcoxon matched-pairs signed rank test; **** *p* < 0.0001; *** *p* < 0.001.

Subsequently, microbiomic data were used to identify the bacterial taxonomic groups affected by the probiotic treatment. To this aim, 16S rRNA gene profiling data were normalized by DESeq2 binomial distribution and then statistically analyzed with Welch’s t-test to compare different time points; six comparisons were performed: T0 vs. T3, T0 vs. T14, T0 vs. T28, T3 vs. T14, T3 vs. T28, and T14 vs. T28. In addition to OTUs, the main taxonomic levels (phylum, class, order, family and genus) were considered in the analysis. The obtained results revealed that the probiotic intervention significantly modified the relative abundance of 3 OTUs, one ascribed to the genus *Lactobacillus*, another to the genus *Bifidobacterium*, and the third to an undefined taxon of the family Lachnospiraceae (Figure 3A). In particular, OTU nr. 34342, taxonomically assigned to the species *Bifidobacterium animalis* and plausibly corresponding to strain *B. animalis* subsp. *lactis* Bl-04 administered with the probiotic formulation, resulted in a significant increase after approximately 3 and 14 days of probiotic intake compared to the level at baseline and T28 (Figure 4). In addition, a few taxa significantly increased after the follow-up period: the phylum Proteobacteria, the classes γ-Proteobacteria and Bacilli, the order Lactobacillales, and the genera *Prevotella* and *Haemophilus* (T28) (Figure 3A).

DESeq2 normalization considers the value “0” for taxa that have not been found in a sample. Nonetheless, since 16S rRNA profiling data generated by NGS are compositional data, the read count should be associated with a probability, which cannot be 1 or 0. To overcome this potential drawback of DESeq2 normalization applied to (meta) genomic/transcriptomic data, an ALDEx2 multivariate probability distribution was proposed [25]. Therefore, subsequently, we performed ALDEx2 normalization of sequencing read abundances followed by Welch t-statistics, obtaining a significant modification over the trial only for the taxonomic levels associated with the *B. animalis* OTU 4426298 (Figure 3B).

Overall, these results indicate that the administration of the probiotic product had a limited effect on the relative abundance of specific fecal bacterial taxa, essentially resulting in the increase in a single OTU ascribed to the species *Bifidobacterium animalis*.

### 3.4. Impact of Probiotic Intervention on the Fecal Levels of Organic Acids

**Figure 5:**
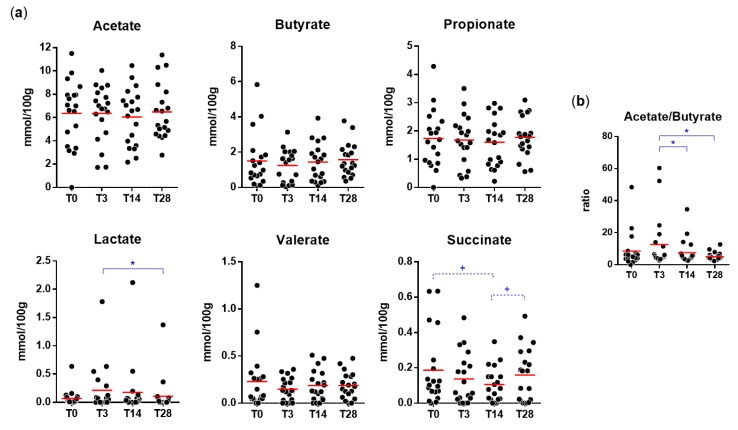
Concentration of short-chain fatty acids, lactate, and pyruvate in fecal samples collected during the trial. Statistically significant differences are according to a paired Student’s *t*-test (* *p* < 0.05; +, *p* between 0.05 and 0.1). (**a**) Absolute concentration; (**b**) acetate/butyrate ratio.

As expected, acetate, propionate, and butyrate were found to be the most abundant SCFAs in fecal samples. Succinate, valerate, and lactate were also detected and quantified (Figure 5). The probiotic intervention did not significantly affect the fecal levels of SCFAs. Nonetheless, we observed a significant increase in lactate levels at T3 and a trend toward decreased succinate at T14. In addition, we also found a significant increase in the acetate/butyrate ratio at T3 compared to the levels at T14 and T28 (Figure 5).

Finally, Spearman’s rho test revealed that the relative abundance of OTU 4426298 at T3 correlated positively with the acetate/butyrate ratio (Spearman’s ρ = 0.389; *p* = 0.0338). At T3, a trend for a negative association between OTU 4426298 and succinate was also observed (Spearman’s ρ = −0.344; *p* = 0.0629). Conversely, these associations were not found at T14.

## 4. Discussion

The “tradition of use” represents a primary aspect to consider for the assessment of the safety of a food microorganism. Accordingly, the evaluation of a history of safe use is a main pillar of the qualified presumption of safety (QPS) concept developed by the European Food Safety Authority (EFSA) to find evidence in support of a “reasonable certainty of no harm” for any microorganism intentionally added to food and feed [26]. The “traditional use” is also explicitly mentioned in the guidelines on probiotics of the Italian Ministry of Health, which stated that “the microorganisms that may be used in food products and food supplements” should have “long been used to supplement human intestinal microflora (microbiota)” [27]. Bifidobacteria, particularly *B. animalis* subsp. *lactis*, have been used in probiotic foods and supplements for more than 20 years; therefore, these bacteria surely fall within the concept of tradition of safe use. Nonetheless, the administration of these bacteria in a quantity that may exceed 50 billion per single dose has become common only in recent years. An experimental confirmation that the intake of bifidobacterial strains can also be safe at higher concentrations is, therefore, warranted.

The intestinal microbiota of healthy adults is characteristically resistant and resilient, i.e., capable of maintaining or re-establishing its taxonomic structure and ecosystem, respectively, upon press or pulse perturbations [28]. Nonetheless, the gut microbiota in urbanized populations is inherently less resilient, plausibly as a consequence of the progressive loss of taxonomic diversity that occurred during the westernization of human societies [29], resulting in the contraction of the “threshold of no return”, i.e., the latitude of taxonomic fluctuations that permits the return to the initial state of the microbial community structure [28]. We can speculate that the intake of probiotic products with a high concentration of live cells could have a greater impact on the taxonomic structure of low-resilience gut microbial ecosystems, such as those of westernized healthy adults. However, this hypothesis has never been experimentally tested.

In the context described above, the primary aim of this study was the characterization of the effect of a probiotic blend containing a high number (i.e., more than 50 billion) of viable bifidobacterial cells on the fecal microbial ecosystem of healthy adults. Specifically, we studied a probiotic formulation with a nominal concentration of 70 billion bacterial CFUs per capsule, 25% of which was composed of three *Lactobacillus* strains (*L. acidophilus* La-14, *L. plantarum* SDZ-11, and *L. paracasei* SDZ-22) and 75% of which was composed of the strain *Bifidobacterium animalis* subsp. *lactis* Bl-04. The intervention in our project consisted of the administration of one probiotic capsule per day on an empty stomach, resulting, therefore, in the intake of at least 52.5 billion CFUs of bifidobacterial cells, which is much greater than the quantity of bifidobacteria that can be consumed in conventional foods. Then, in this study, we assessed the “microbiomic safety” of the product (i.e., the potential consequences of its consumption on the intestinal microbial ecosystem) by analyzing the feces of healthy adult volunteers recruited in a highly urbanized European area (Milan metropolitan area, Italy). Specifically, the impact of the intake of the probiotic formulation on the intestinal microbial ecosystem was assessed by 16S rRNA gene profiling and SCFA quantification in fecal samples collected over the course of 4 weeks from 19 healthy adult volunteers who ingested one capsule per day for 2 weeks. Similar studies have been carried out with the popular probiotic product VSL#3, a blend of several bacterial strains, which has a large amount of scientific literature attesting to its clinical efficacy. VSL#3 has a nominal concentration of 450 billion CFUs per dose, 60% of which was composed of *Streptococcus thermophilus* and approximately 30% of which was composed of *B. animalis* subsp. *lactis* (i.e., at least 135 billion bifidobacterial CFUs per dose) [30]. In the study by Michail and Kenche, 24 subjects with diarrhea-predominant IBS consumed 2 doses per day of VSL#3 for 8 weeks (i.e., nominally, 900 billion viable bacterial cells per day, at least 270 billion of which were represented by bifidobacteria). Although authors found improvement in specific Gastrointestinal Symptom Rating Scale (GSRS) scores, the fecal microbiota composition, assessed using phylogenetic microarray, was not significantly affected [31]. More recently, the administration of VSL#3 to 14 healthy women (two capsules a day for 4 weeks, each capsule containing at least 112.5 billion viable bacterial cells) led to a significant reduction in the relative number of circulating T helper 17 lymphocytes; nonetheless, the abundance of fecal bacterial taxa (investigated through 16S rRNA gene profiling) and α-diversity did not change following administration of the probiotic [32]. These studies indicate that the intake of probiotic bacteria (including bifidobacteria) at doses of hundreds of billions of organisms is safe and suggest that the mechanism of action of such probiotic products is plausibly not directly linked to the microbiota.

Similarly, the results of our study indicate that the intake of a high number of viable bifidobacterial cells did not drastically affect the bacterial taxonomic composition in the feces of healthy adult volunteers. Nonetheless, we observed a significant transient increase in the bifidobacteria introduced by the probiotic. The bifidobacterial strain in the probiotic product under study, named *B. animalis* subsp. *lactis* Bl-04, has extensive literature attesting to its properties, particularly in terms of immunomodulation [33,34,35,36]. The modulation of immune responses by microorganisms in the gut is mainly exerted in the ileum, where most of the intestinal structures of the mucosa-associated lymphoid tissue (MALT) are located [37]. We can speculate that the localized action of the probiotic at the ileum level could justify the limited influence on the bacterial community structure observed in feces.

Immunomodulation is promoted by the direct contact of microorganisms with the intestinal mucosa, which is reasonably favored when the concentration of a specific microorganism is increased. It has been calculated that, after the ingestion of 10 billion cells of a probiotic, assuming a 10% survival rate during gastrointestinal transit, the relative proportion of ingested bacteria can reach a level of 0.01- to 1-fold that of the resident bacteria in the ileum, therefore transiently becoming a dominant member of the microbiota at that specific site, having the opportunity to interact directly with the host [38]. Therefore, the intake of a larger amount of a probiotic strain with immunomodulatory properties, such as *B. animalis* subsp. *lactis* Bl-04, might plausibly boost the establishment of a dialogue with the ileal mucosa of the host.

Bifidobacteria are anaerobic fermentative bacteria that catabolize carbon sources into acetate and lactate at a 1.5:1 ratio. Accordingly, during the probiotic intervention, we found an increase in fecal lactate levels and the acetate/butyrate ratio. Specifically, the observed changes in fecal organic acid concentrations were mainly observed at T3, i.e., a few days after the beginning of the probiotic intake period, but returned to the initial levels at the end of the probiotic treatment (T14). Notably, correlation analyses showed a positive association between the fecal levels of the *B.* animalis OTU 4426298 and the acetate/butyrate ratio ad T3 but not at T14. These results suggest that the perturbation induced by the intake of the probiotic product exerts its effect mostly during the first days whereas, subsequently, it could be efficiently coped with by the resilience mechanisms of the gut microbiota of the healthy adults participating in the trial. Furthermore, upon probiotic intake, we also observed a trend toward a decrease in the fecal levels of succinate, a microbial product resulting from intestinal fermentation of dietary fiber. An increase in intestinal succinate concentrations has been associated with dysbiosis and enhanced inflammatory processes in the gut [39]; therefore, interventions that can reduce this organic acid in the intestine can plausibly benefit the host.

In conclusion, this study indicates that the administration of the bifidobacterial strain Bl-04 at a high dose through the administration of the investigated multistrain probiotic formulation (which also included three different species of lactobacilli) was well tolerated by the intestinal microbial ecosystem of healthy adult volunteers. In addition, the observed transient increase in fecal bifidobacteria, lactic acid, and the acetate/butyrate ratio may contribute to the implementation of mechanisms that are widespread among probiotics, such as colonization resistance and the competitive exclusion of pathogens.

## References

[1] Gibson G.R., Hutkins R., Sanders M.E., Prescott S.L., Reimer R.A., Salminen S.J., Scott K., Stanton C., Swanson K.S., Cani P.D. (2017). Expert consensus document: The international scientific association for probiotics and prebiotics (isapp) consensus statement on the definition and scope of prebiotics. Nat. Rev. Gastroenterol. Hepatol..

[2] Wilkins T., Sequoia J. (2017). Probiotics for gastrointestinal conditions: A summary of the evidence. Am. Fam. Physician.

[3] Ouwehand A.C. (2017). A review of dose-responses of probiotics in human studies. Benef. Microbes.

[4] Johnston B.C., Goldenberg J.Z., Vandvik P.O., Sun X., Guyatt G.H. (2011). Probiotics for the prevention of pediatric antibiotic-associated diarrhea. Cochrane Database Syst. Rev..

[5] McFarland L.V. (2006). Meta-analysis of probiotics for the prevention of antibiotic associated diarrhea and the treatment of clostridium difficile disease. Am. J. Gastroenterol..

[6] Taverniti V., Koirala R., Dalla Via A., Gargari G., Leonardis E., Arioli S., Guglielmetti S. (2019). Effect of cell concentration on the persistence in the human intestine of four probiotic strains administered through a multispecies formulation. Nutrients.

[7] Hidalgo-Cantabrana C., Delgado S., Ruiz L., Ruas-Madiedo P., Sanchez B., Margolles A. (2017). Bifidobacteria and their health-promoting effects. Microbiol. Spectr..

[8] Milani C., Duranti S., Lugli G.A., Bottacini F., Strati F., Arioli S., Foroni E., Turroni F., van Sinderen D., Ventura M. (2013). Comparative genomics of bifidobacterium animalis subsp. Lactis reveals a strict monophyletic bifidobacterial taxon. Appl. Environ. Microbiol..

[9] Miller L.E., Ouwehand A.C. (2013). Probiotic supplementation decreases intestinal transit time: Meta-analysis of randomized controlled trials. World J. Gastroenterol..

[10] Chmielewska A., Szajewska H. (2010). Systematic review of randomised controlled trials: Probiotics for functional constipation. World J. Gastroenterol..

[11] Dimidi E., Christodoulides S., Fragkos K.C., Scott S.M., Whelan K. (2014). The effect of probiotics on functional constipation in adults: A systematic review and meta-analysis of randomized controlled trials. Am. J. Clin. Nutr..

[12] Airaksinen K., Yeung N., Lyra A., Lahtinen S.J., Huttunen T., Shanahan F., Ouwehand A.C. (2019). The effect of a probiotic blend on gastrointestinal symptoms in constipated patients: A double blind, randomised, placebo controlled 2-week trial. Benef. Microbes.

[13] Hempel S., Newberry S.J., Maher A.R., Wang Z., Miles J.N., Shanman R., Johnsen B., Shekelle P.G. (2012). Probiotics for the prevention and treatment of antibiotic-associated diarrhea: A systematic review and meta-analysis. JAMA.

[14] McFarland L.V., Evans C.T., Goldstein E.J.C. (2018). Strain-specificity and disease-specificity of probiotic efficacy: A systematic review and meta-analysis. Front. Med..

[15] Miller L.E., Lehtoranta L., Lehtinen M.J. (2017). The effect of bifidobacterium animalis ssp. Lactis hn019 on cellular immune function in healthy elderly subjects: Systematic review and meta-analysis. Nutrients.

[16] Eales J., Gibson P., Whorwell P., Kellow J., Yellowlees A., Perry R.H., Edwards M., King S., Wood H., Glanville J. (2017). Systematic review and meta-analysis: The effects of fermented milk with bifidobacterium lactis cncm i-2494 and lactic acid bacteria on gastrointestinal discomfort in the general adult population. Ther. Adv. Gastroenterol..

[17] Isolauri E., Rautava S., Salminen S. (2012). Probiotics in the development and treatment of allergic disease. Gastroenterol. Clin. North Am..

[18] Watanabe K., Makino H., Sasamoto M., Kudo Y., Fujimoto J., Demberel S. (2009). Bifidobacterium mongoliense sp. Nov., from airag, a traditional fermented mare’s milk product from mongolia. Int. J. Syst. Evol. Microbiol..

[19] Cattaneo C., Gargari G., Koirala R., Laureati M., Riso P., Guglielmetti S., Pagliarini E. (2019). New insights into the relationship between taste perception and oral microbiota composition. Sci. Rep..

[20] Caporaso J.G., Kuczynski J., Stombaugh J., Bittinger K., Bushman F.D., Costello E.K., Fierer N., Pena A.G., Goodrich J.K., Gordon J.I. (2010). Qiime allows analysis of high-throughput community sequencing data. Nat. Methods.

[21] Gargari G., Taverniti V., Balzaretti S., Ferrario C., Gardana C., Simonetti P., Guglielmetti S. (2016). Consumption of a bifidobacterium bifidum strain for 4 weeks modulates dominant intestinal bacterial taxa and fecal butyrate in healthy adults. Appl. Environ. Microbiol..

[22] Gargari G., Taverniti V., Gardana C., Cremon C., Canducci F., Pagano I., Barbaro M.R., Bellacosa L., Castellazzi A.M., Valsecchi C. (2018). Fecal clostridiales distribution and short-chain fatty acids reflect bowel habits in irritable bowel syndrome. Environ. Microbiol..

[23] Love M.I., Huber W., Anders S. (2014). Moderated estimation of fold change and dispersion for rna-seq data with deseq2. Genome Biol..

[24] Fernandes A.D., Reid J.N., Macklaim J.M., McMurrough T.A., Edgell D.R., Gloor G.B. (2014). Unifying the analysis of high-throughput sequencing datasets: Characterizing rna-seq, 16s rrna gene sequencing and selective growth experiments by compositional data analysis. Microbiome.

[25] Fernandes A.D., Macklaim J.M., Linn T.G., Reid G., Gloor G.B. (2013). Anova-like differential expression (aldex) analysis for mixed population rna-seq. PLoS ONE.

[26] EFSA (2007). Introduction of a qualified presumption of safety (qps) approach for assessment of selected microorganisms referred to efsa. EFSA J..

[27] Mohajeri M.H., Brummer R.J.M., Rastall R.A., Weersma R.K., Harmsen H.J.M., Faas M., Eggersdorfer M. (2018). The role of the microbiome for human health: From basic science to clinical applications. Eur. J. Nutr..

[28] Sommer F., Anderson J.M., Bharti R., Raes J., Rosenstiel P. (2017). The resilience of the intestinal microbiota influences health and disease. Nat. Rev. Microbiol..

[29] Segata N. (2015). Gut microbiome: Westernization and the disappearance of intestinal diversity. Curr. Biol. CB.

[30] Mora D., Filardi R., Arioli S., Boeren S., Aalvink S., de Vos W.M. (2019). Development of omics-based protocols for the microbiological characterization of multi-strain formulations marketed as probiotics: The case of vsl#3. Microb. Biotechnol..

[31] Michail S., Kenche H. (2011). Gut microbiota is not modified by randomized, double-blind, placebo-controlled trial of vsl#3 in diarrhea-predominant irritable bowel syndrome. Probiotics Antimicrob. Proteins.

[32] Singh A., Sarangi A.N., Goel A., Srivastava R., Bhargava R., Gaur P., Aggarwal A., Aggarwal R. (2018). Effect of administration of a probiotic preparation on gut microbiota and immune response in healthy women in india: An open-label, single-arm pilot study. BMC Gastroenterol..

[33] Martinez F.A., Dominguez J.M., Converti A., Oliveira R.P. (2015). Production of bacteriocin-like inhibitory substance by bifidobacterium lactis in skim milk supplemented with additives. J. Dairy Res..

[34] Foligne B., Nutten S., Grangette C., Dennin V., Goudercourt D., Poiret S., Dewulf J., Brassart D., Mercenier A., Pot B. (2007). Correlation between in vitro and in vivo immunomodulatory properties of lactic acid bacteria. World J. Gastroenterol..

[35] Sollid L.M., Brandtzaeg P., Kvale D., Gaudernack G., Scott H., Thorsby E. (1988). T cell-epithelium interactions in relation to gut immunity. Monogr. Allergy.

[36] Paineau D., Carcano D., Leyer G., Darquy S., Alyanakian M.A., Simoneau G., Bergmann J.F., Brassart D., Bornet F., Ouwehand A.C. (2008). Effects of seven potential probiotic strains on specific immune responses in healthy adults: A double-blind, randomized, controlled trial. FEMS Immunol. Med. Microbiol..

[37] Santaolalla R., Fukata M., Abreu M.T. (2011). Innate immunity in the small intestine. Curr. Opin. Gastroenterol..

[38] Derrien M., van Hylckama Vlieg J.E. (2015). Fate, activity, and impact of ingested bacteria within the human gut microbiota. Trends Microbiol..

[39] Connors J., Dawe N., Van Limbergen J. (2018). The role of succinate in the regulation of intestinal inflammation. Nutrients.

